# Subsidies versus intellectual property rights when innovators operate in two markets

**DOI:** 10.1371/journal.pone.0284880

**Published:** 2023-04-24

**Authors:** Egle Skliaustyte, Matthias Weber

**Affiliations:** 1 Center for Excellence in Finance and Economic Research, Bank of Lithuania, Vilnius, Lithuania; 2 School of Finance, University of St. Gallen, St. Gallen, Switzerland; 3 Swiss Finance Institute, St. Gallen, Switzerland; University of Malta, MALTA

## Abstract

Intellectual property rights are monopoly rights, which have undesirable welfare properties. Therefore, several studies suggest using rewards as incentives for innovation instead. However, these studies have thus far had little effect on actual policy, possibly because such rewards may be difficult to implement in practice. We suggest a new way of providing incentives to originators, which is easier to implement. Our suggestion can be used if there is an additional market in which originators operate, where copying is not easily possible. In this case, intellectual property rights in one market can be replaced by subsidies in the other market. Taking the music industry as example, copyrights in the records market could be replaced by subsidies in the market for live performances. We develop a partial equilibrium model that can be used to analyze in which cases the replacement of intellectual property rights in one market with subsidies in another market is welfare improving and better for the originator. A numerical application example suggests that the subsidy scheme may indeed be better in the music industry. The subsidy scheme can be implemented as a voluntary option, which would even be possible without changing the legal framework of intellectual property rights.

## Introduction

There is a longstanding debate on how innovation and creativity should be incentivized. The standard in today’s world is to assign intellectual property rights, which are monopoly rights on an idea or a creative work, awarded to its originator. However, monopolies are in general inefficient, because monopolists set output levels that are lower than the efficient levels of a competitive market (leading to what is called a deadweight loss). Intellectual property rights have thus harmful effects. This has long been known in economics. The harmful effects of intellectual property rights are compellingly described in the book by [1]. In some cases, there are arguments for abolishing intellectual property rights without a substitute (e.g., [2, 3]). However, the standard arguments in favor of intellectual property rights are that there need to be incentives to spur innovation and creativity and that it is only fair if the originators of creative works or innovations receive some sort of monetary remuneration for their work (which may, without intellectual property rights and without other forms of compensation, not accrue, as ideas and works can be copied for free).

Because of the inefficiency of intellectual property rights on the one hand and the wish for compensation for the innovator or artist on the other, scholars have been looking for forms of compensation other than awarding monopoly rights. In several studies, researchers find that rewards or prizes can be alternatives superior to awarding monopoly rights (see the reviewed literature in the following section). Rewards have been shown to improve over intellectual property rights in several situations in different studies, but this research has only had a limited impact on actual policy. A reason could be that such rewards are difficult to implement. It may be relatively easy to show that a government could theoretically do well with assigning rewards to innovations of a certain quality; in practice, the sheer number of innovations and creative works plus the difficulty of assessing their quality make it difficult to use rewards for compensation (of course, also the implementation and enforcement of intellectual property rights is not necessarily easy: complicated law suits and threats thereof impose a burden on society).

For our paper, we exploit the fact that in many innovative or creative industries, originators are not only active in the primary market in which copying is easily possible but also in a secondary market in which copying is difficult or impossible. The two markets are most easily illustrated with the example of the music industry. Music can easily be copied when consumed at home, because CDs and music files on the computer can easily be copied (basically for free). However, musicians do not only earn money from selling CDs and mp3 files. They also earn money from live performances. The market for live performances is the secondary market. In that market, copying is difficult: while there is no difference between listening to an original mp3 file of your favorite song and a copy of this file, there is a big difference between attending a concert of your favorite band and one of a different band playing the songs of your favorite band. Note that only a small fraction of a musician’s income typically stems from the sale of records: only about 6% in the US, while the largest shares of income are live performances with about 28% and teaching with about 22% [4]. The situation is comparable in other countries (e.g., China or Switzerland; [5, 6]). For simplicity, we focus on subsidies in only one other market, but the analysis could easily be extended to multiple other markets (for instance, removing copyrights in the music records market and introducing subsidies for concerts and for music teaching).

Similar to a part of the existing literature, we suggest using subsidies instead of intellectual property rights (in some cases). However, we do not suggest using subsidies in the same market in which innovation takes place and in which copying is easily possibly. Instead, we suggest implementing subsidies in the secondary market in which originators are active, in which copying is not easily possible. What we provide in this paper is a simple analytical framework based on economic partial-equilibrium modeling. This framework can be used to assess whether the introduction of a subsidy in the second market, in exchange for a removal of intellectual property rights in the first market, leads to an improvement in social welfare and to a pareto improvement (meaning that everyone, including the originator, is better off). After providing the framework and deriving the theoretical results that can be used for such a comparison, we show an application example with a parameter calibration that seems suitable for the music industry, suggesting that the (possibly voluntary) subsidy scheme in the market for live performances may indeed improve over intellectual property rights in the records market.

The main difference of our model and our policy implications to the literature is the following. While subsidies or rewards are often superior to intellectual property rights in the literature, these are always subsidies or rewards in the same market in which the innovation takes places. The use of the secondary market in which the originators operate is unique to our study.

The secondary market allows for subsidies that can be easily implemented to compensate the originator for not receiving monopoly rights in the first market. The reason why subsidies cannot be implemented easily in the primary market is that one would need a measure of the quality of the innovation in that market after intellectual property rights have been removed. This is difficult to obtain: in the absence of monopoly rights to the originator, the government does not have a market price to base the subsidy on (because copying is possible at no or low marginal cost). In the secondary market, this problem does not exist, because copying is not easily possible and the sales in that market are observed.

The simplest implementation of a subsidy is probably the reduction of a tax. With value added tax (VAT) rates of about 20–25% in many countries worldwide (especially in Europe), applying a reduced VAT rate in the second market is an extremely easy and efficient way to implement such a subsidy (reduced VAT rates often exist for some product categories anyways, for instance for food). In practice, there is a difference between tax breaks and other forms of subsidies where the government transfers money (e.g., a difference concerning the implied bureaucracy and the opportunity for fraud), but theoretically, they are alike: both cases constitute a transfer of money from the government to the recipient. Consequently, the model shown in this paper does not distinguish between the two; we simply refer to both as a subsidy most of the time. Note that in the EU, such subsidies would not be considered state aid, as they would be available to all individuals and companies.

The replacement of intellectual property rights in one market with a subsidy in the other market would not necessarily have to be forced upon everyone in an industry. One could also consider a policy where this happens on a voluntary basis. For instance, musicians could opt into a scheme that grants them subsidies on life performances. Opting into this scheme would only be possible for musicians who make their music available without copyrights in the records market. Such a voluntary version could be particularly popular, because both consumers and artists/producers would be made better off, while the owners of already existing copyrights would hardly be affected. Such a voluntary scheme could also be implemented without changing the legal framework on the protection of intellectual property.

For applications, we like to think first and foremost about situations in which intellectual property rights are assigned as copyrights, such as music and movies, in particular music. This makes things simple, as the produced works are final consumption goods rather than production inputs and as obvious secondary markets exist (music live performances and movies shown in cinemas, respectively). For movies, one could eliminate copyrights for CDs and digital files and compensate movie companies by subsidizing the screening in cinemas (to be precise, one would not completely eliminate copyrights for movies: instead, copyrights would only be removed for non-commercial use, so that cinemas would still have to pay for the movies).

However, there are also other cases in the area of intellectual property rights, with second or third markets. Researchers, for instance, publish articles that can easily be copied and distributed, but they also make money by teaching at universities and similar institutions, with the pay for their teaching often positively correlated with their research output. Forgetting for a moment about the fact that it is in general publishers and not the authors themselves who hold the copyrights of the articles, it would be possible to eliminate copyrights on scientific articles and instead subsidize teaching in higher education, or increase such subsidies if they are already in place for other reasons (in the case of scientific publishing, it seems that those who profit the most from copyright are not the originators but the publishers: there are arguments that this is not an exception, but that the introduction of copyrights in general mainly benefits intermediaries and not originators, see [7]).

Secondary markets (with activity or sales complementary to those in the primary copyright-protected market) do not need to exist in all areas. In areas where such markets exist, it is not clear that a subsidy in the second market is better than granting monopoly rights in the first market. For instance, authors of books make money in public readings in addition to the money they receive from the selling of the books, but the secondary market of public readings seems too small to be useful as compensation for removing copyrights in the book market. Each area needs to be assessed by itself. In the music market, the fact that money from the records market only makes up for a small fraction of a musician’s income makes it likely that moderate subsidies in the market for live music would make everyone better off, including the musicians.

## Related literature

Intellectual property rights have socially undesirable welfare properties (the deadweight loss). These inefficiencies may sometimes be overcome with different mechanisms of providing incentives or rewards to originators. Therefore, an interesting literature developed comparing subsidies, rewards, and prizes to intellectual property rights theoretically, since at least [8]. This author compares patents, direct contracting for research, and prizes. He finds that intellectual property rights are inferior except in a part of the situations with an imbalance of information about costs and benefits of the innovation. [9] compare reward systems and intellectual property rights in a model with endogenous effort provision by the innovator. They find that an optional reward system is always superior to an intellectual property right system. A compulsory reward system may or may not be better than the patent system or than the optional reward system. The work by [10] proposes a new prize system for the innovation of pharmaceuticals, which would improve allocative efficiency over the patent system and remove the deadweight loss arising from patents. This prize system is based on value-based pricing and exploits the fact that some information about health benefits can be based on the costs of palliative or nursing care. A dynamic model for the research and development of new drugs with patents or an alternative reward system is developed in [11]. The alternative reward system that these authors propose, which they call intertemporal bounty, is a payment to the innovating firm, determined as a fraction of all sales of the innovative product by all producers (thus not only by the sales of the product by the innovating firm). They show that this system can increase welfare substantially as compared to a patent system. [12] compare prizes and patents in a model in which the government cannot observe the quality of the produced good. Yet, signals obtained from other market participants can be used to infer the quality of the good. In this model, prizes are superior to patents if the innovator cannot manipulate the market signals that the government receives (while patents are superior if the innovator can manipulate these signals costlessly).

Part of the literature analyzes the effects of abandoning intellectual properties without replacing them by a different reward system. [2] develop a model showing that societies may be better off without patent protection than with it when innovation is sequential and complementary; that is, when successive innovations build on the work by predecessors and when all potential innovators take different research lines. They connect their model to the innovative time in the software industry, when patent protection was weak. In a model of the music industry with superstars and regular artists, [13] show that copyrights in the music industry have ambiguous effects. On the one hand, large revenues of superstars are incentives for young artists to start a career, on the other hand, the consequential large market shares of superstars make the entry and survival of young artists more difficult.

There is also a strand of the literature comparing intellectual property rights to alternative reward systems or analyzing the effectiveness of such systems not making use of (formal) theoretical models. [14], for instance, argue for the replacement of intellectual property rights with alternative incentive and reward systems based based on recent trends in the innovation landscape. Empirical evidence that abandoning or weakening intellectual property rights can be beneficial is provided by [3], who exploit an exogenous shift to weak copyrights during the second world war. [15] show empirically that prizes offered by the Royal Agricultural Society of England between 1839 and 1939 where successful in spurring innovation.

The state of knowledge and the literature on incentive systems for innovation are discussed and reviewed in [16–18]. As [18] states, “there are strong theoretical reasons that an ideal prize or reward system could dominate an ideal patent system because of the reduction of deadweight loss”, but he also cautions that “arguments for and against prize and reward systems are many and all depend on empirical considerations”.

Our research fits most closely with the literature providing theoretical models analyzing in which cases subsidies, rewards, or prizes are superior to intellectual property rights. As already mentioned in the introduction, the fundamental difference of our study with respect to this theoretical literature is that the other articles analyze when alternative incentive schemes *in the same market where the innovation takes place* improve over intellectual property rights in that market. Making use of the fact that originators are active in more than one market, and analyzing the effect of subsidies in this secondary market instead of awarding intellectual property rights, is absolutely novel. With our way of modeling (using relatively simple partial equilibrium modeling with linear equations), we provide a framework reacting to the need of models that are suitable for empirical work (as called for in the above quote by [18]). This framework can then be used to assess which ways of incentivizing innovators are best in a given real-world market.

## Method and derivations

The method we use is partial equilibrium modeling, a standard way of building theoretical models in economics. With the model that we are building, we can then compare the welfare properties and producer surpluses under the two policy regimes (two cases). We resort to linear equations throughout the paper for simplicity. In applications to data, it may be easiest to estimate linear equations—the model could also be extended to non-linear equations, but this would make the model less tractable.

We first develop the model for the status quo (case 1), with intellectual property rights in the first market (in which the innovation takes place) and without a subsidy in the second market. There is a monopoly in both markets: due to the assignment of intellectual property rights in the first market and naturally in the second market. Thereafter, we adjust the model to the second policy regime (case 2). In that case, the intellectual property rights in the first market are removed, while a subsidy is introduced in the second market. In case 2, there is perfect competition in the first market (everyone can copy the product), while there is a subsidized monopoly in the second market.

### Both markets operating as monopolies (Status Quo, Case 1)

There are two markets with two goods. The first market is the market in which innovation takes place, where works could technically easily be copied. In the second market copying is not possible. Both markets operate as monopolies (due to the intellectual property rights in the first market, naturally in the second) and no subsidy is provided.

#### First market—Monopoly

**Fig 1:**
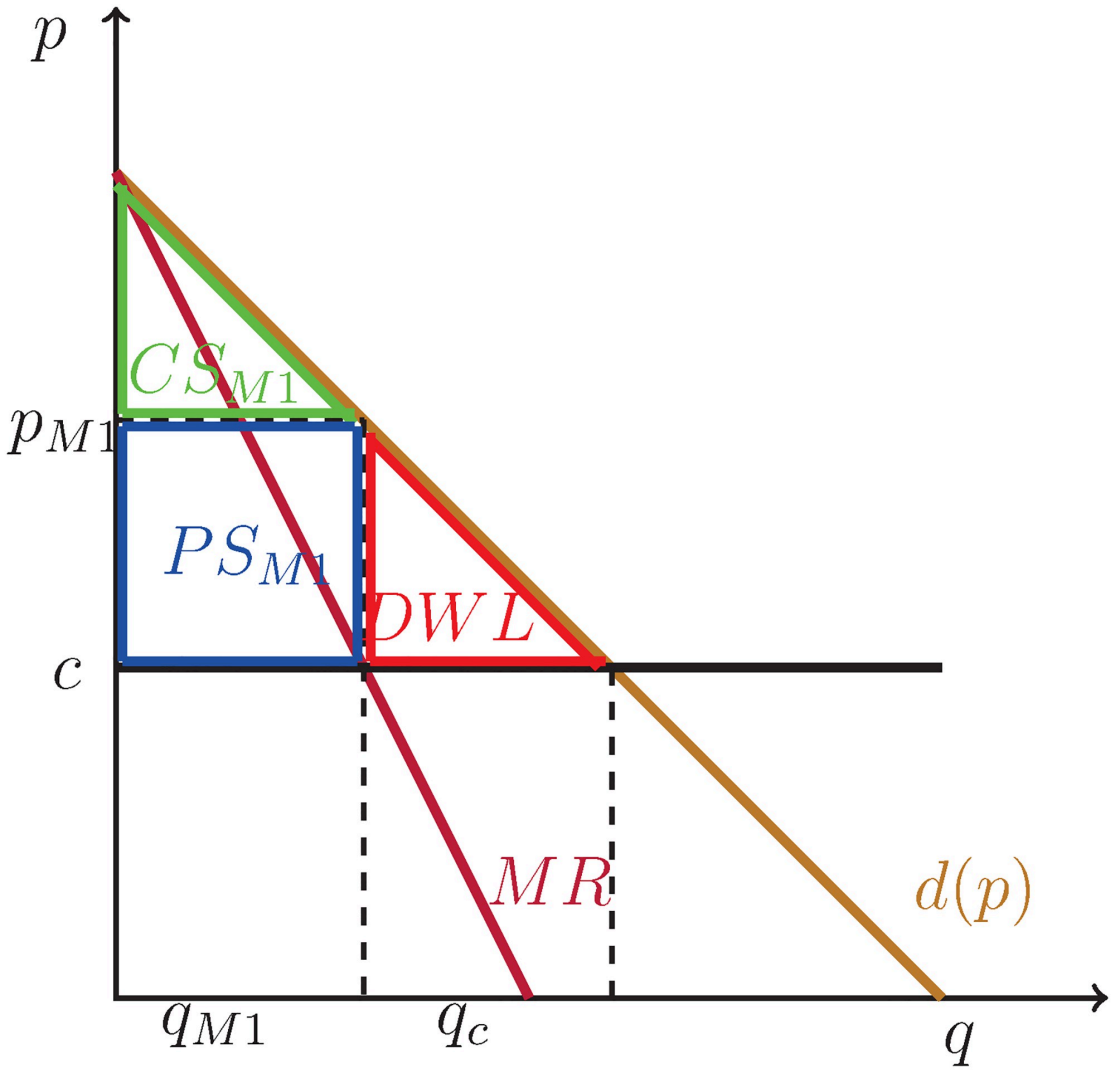
Case 1, market 1: Monopoly with constant marginal cost.

First, consider the structure of the first market, in which the innovation takes place. The market is illustrated graphically in Fig 1. The marginal cost is constant at *c* ≥ 0 and is thus represented by a horizontal line. This marginal cost can be thought of as the cost of copying the idea (*c* may be small, e.g. when copying CDs, or even approximately zero, e.g. when copying mp3 files).

The demand for the innovation product and the marginal revenue are given by the following equations, with positive parameters *ψ* and *ϕ* (to be precise, the equation is the equation of the inverse demand curve, as demanded quantity is a function of price—as supply-demand graphs are typically drawn with quantity on the horizontal axis, we will simply refer to these equations as demand equations in the text):

$$\begin{array}{cc} D(q):p & =\psi -\phi q\\ MR(q) & =\psi -2\phi q. \end{array}$$

The profit maximizing choice for the innovator/monopolist is to operate where the marginal revenue is equal to marginal cost:

$$\begin{array}{cc} \psi -2\phi q & =c\\ \;q_{M1} & =\frac{\psi -c}{2\phi }. \end{array}$$

The price charged by the originator, who acts as monopolist due to the intellectual property rights, can be obtained by entering the monopoly quantity into the equation for the demand curve:

$$p_{M1}=\psi -\phi q_{M1}=\psi -\phi \frac{\psi -c}{2\phi }=\frac{\psi +c}{2}.$$

The producer surplus is calculated as the difference between the price and the cost multiplied by the monopoly quantity:

$$PS_{M1}=\left(p_{M1}-c\right)q_{M1}=\left(\frac{\psi +c}{2}-c\right)\left(\frac{\psi -c}{2\phi }\right)=\frac{{\left(\psi -c\right)}^{2}}{4\phi }.$$

The consumer surplus (the triangle below the demand curve and above the monopoly price *p*_*M*1_) is thus:

$$CS_{M1}=\frac{\psi -p_{M1}}{2}q_{M1}=\left(\frac{\psi -\frac{\psi +c}{2}}{2}\right)\left(\frac{\psi -c}{2\phi }\right)=\frac{{\left(\psi -c\right)}^{2}}{8\phi }.$$

There is a deadweight loss resulting from the monopoly structure marked as DWL in the graph. It reduces total welfare when compared to perfect competition. Total welfare in this innovation market is thus the sum of the producer surplus and the consumers surplus:

$$\begin{array}{c} TW_{M1}=PS_{M1}+CS_{M1}=\frac{3{\left(\psi -c\right)}^{2}}{8\phi }. \end{array}$$

#### Second market—Monopoly

**Fig 2:**
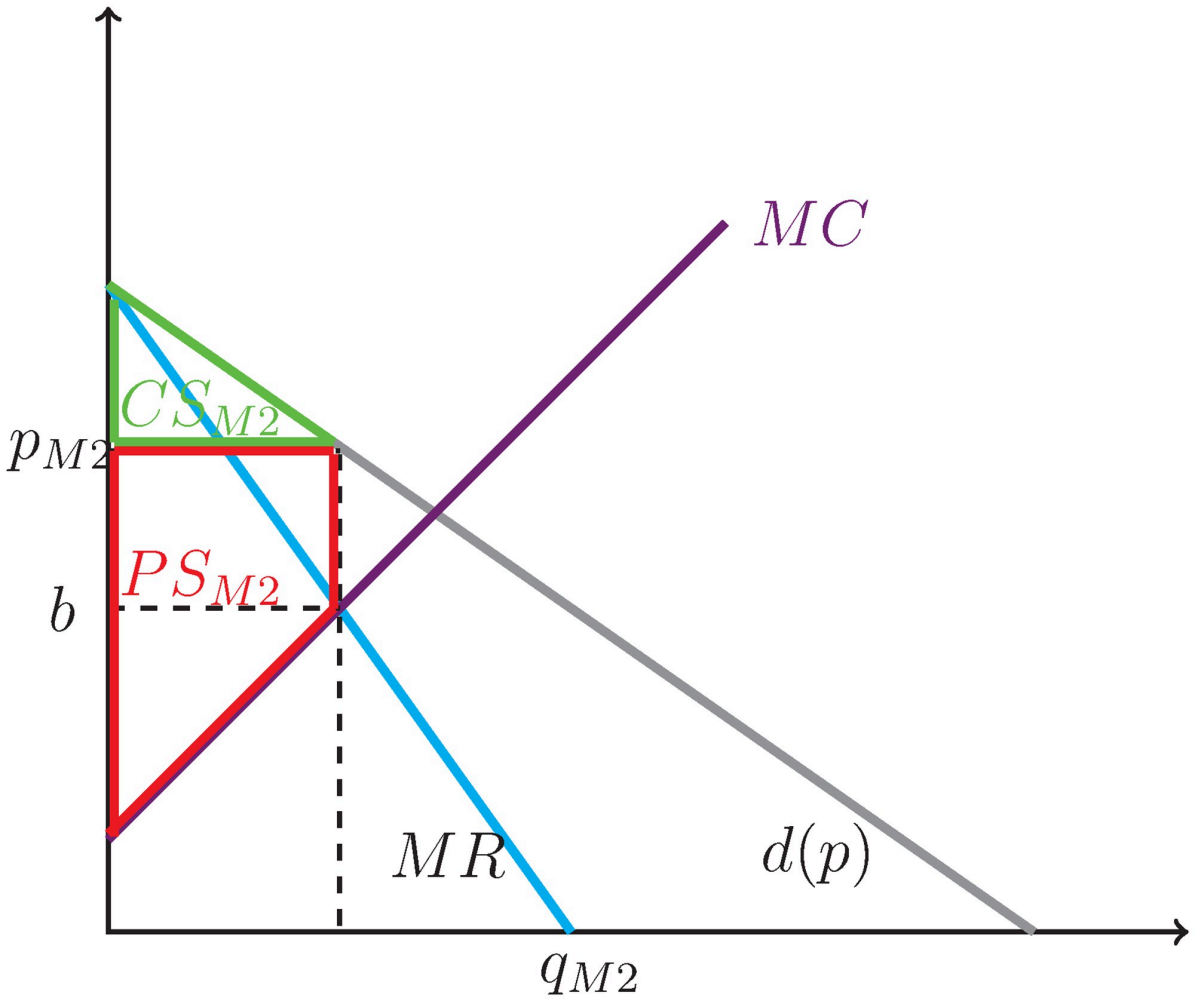
Case 1, market 2: Monopoly with increasing marginal cost.

In the second market, the originator also has monopolistic power (however, this time naturally and not due to the assignment of any rights; in the music example, this corresponds to giving concerts) and operates at an increasing marginal cost: in the music example, each additional concert is costly for the artist. This market is illustrated in Fig 2.

The equations for marginal costs, demand, and marginal revenue are:

$$\begin{array}{cc} MC(q) & =\theta +\epsilon q\\ D(q):p & =\gamma -\eta q\\ MR(q) & =\gamma -2\eta q. \end{array}$$

The optimal quantity of production for the monopolist is then as follows:

$$\begin{array}{cc} MC & =MR\\ \theta +\epsilon q_{M2} & =\gamma -2\eta q_{M2}\\ \epsilon q_{M2}+2\eta q_{M2} & =\gamma -\theta \\ q_{M2} & =\frac{\gamma -\theta }{\epsilon +2\eta }. \end{array}$$

To calculate the producer surplus, the price at which marginal revenue equals marginal cost is needed, which we denote by *b*:

$$\begin{array}{cc} b & =\gamma -2\eta q_{M2}\\ b & =\gamma -2\eta (\frac{\gamma -\theta }{\epsilon +2\eta }). \end{array}$$

Furthermore, the price charged by the monopolist is:

$$p_{M2}=\gamma -\eta q_{M2}=\gamma -\eta \frac{\gamma -\theta }{\epsilon +2\eta }.$$

The consumer surplus (the triangle below the demand curve and above the price *p*_*M*2_ is:

$$\begin{array}{c} CS_{M2}=\frac{\gamma -p_{M2}}{2}q_{M2}=\frac{\gamma -(\gamma -\eta *\frac{\gamma -\theta }{\epsilon +2\eta })}{2}\left(\frac{\gamma -\theta }{\epsilon +2\eta }\right)=\frac{\eta }{2}{\left(\frac{\gamma -\theta }{\epsilon +2\eta }\right)}^{2}. \end{array}$$

The producer surplus (the area from 0 to the optimum quantity *q*_*M*_ below *p*_*M*2_ and above the marginal cost curve) is:

$$\begin{array}{cc} PS_{M2} & =\left(p_{M2}-b\right)q_{M2}+\frac{b-\theta }{2}q_{M2}=q_{M2}\left(p_{M2}-\frac{b}{2}-\frac{\theta }{2}\right)\\ & =\left(\frac{\gamma -\theta }{\epsilon +2\eta }\right)\left(\left(\gamma -\eta \frac{\gamma -\theta }{\epsilon +2\eta }\right)-\frac{\gamma -2\eta (\frac{\gamma -\theta }{\epsilon +2\eta })}{2}-\frac{\theta }{2}\right)=\frac{{\left(\gamma -\theta \right)}^{2}}{2(\epsilon +2\eta )}. \end{array}$$

The total welfare for this market is then:

$$\begin{array}{cc} TW_{M2} & =PS_{M2}+CS_{M2}=\left(\frac{\gamma -\theta }{\epsilon +2\eta }\right)\left(\frac{\gamma +\left(\gamma -\eta \frac{\gamma -\theta }{\epsilon +2\eta }\right)-\left(\gamma -2\eta \left(\frac{\gamma -\theta }{\epsilon +2\eta }\right)\right)-\theta }{2}\right)\\ & =\frac{\gamma -\theta }{\epsilon +2\eta }*\frac{\gamma +\eta \frac{\gamma -\theta }{\epsilon +2\eta }-\theta }{2}=\frac{{\left(\gamma -\theta \right)}^{2}}{2(\epsilon +2\eta )}\left(1+\frac{\eta }{\epsilon +2\eta }\right). \end{array}$$

### Subsidy in the second market instead of monopoly rights in the first (Subsidy Regime, Case 2)

In this setting, there is no monopoly in the first market: the first good is produced with a constant marginal cost *c* and operates in a competitive market (the intellectual property rights have been removed, therefore anyone can copy the good, e.g. the CDs, at constant marginal cost). The second market (with increasing marginal costs) still operates as a monopoly and now receives a government subsidy. The innovator receives the subsidy in the second market as an incentive or compensation, because in the first market with perfect competition there are no profits for the originator.

#### First market—Competition

**Fig 3:**
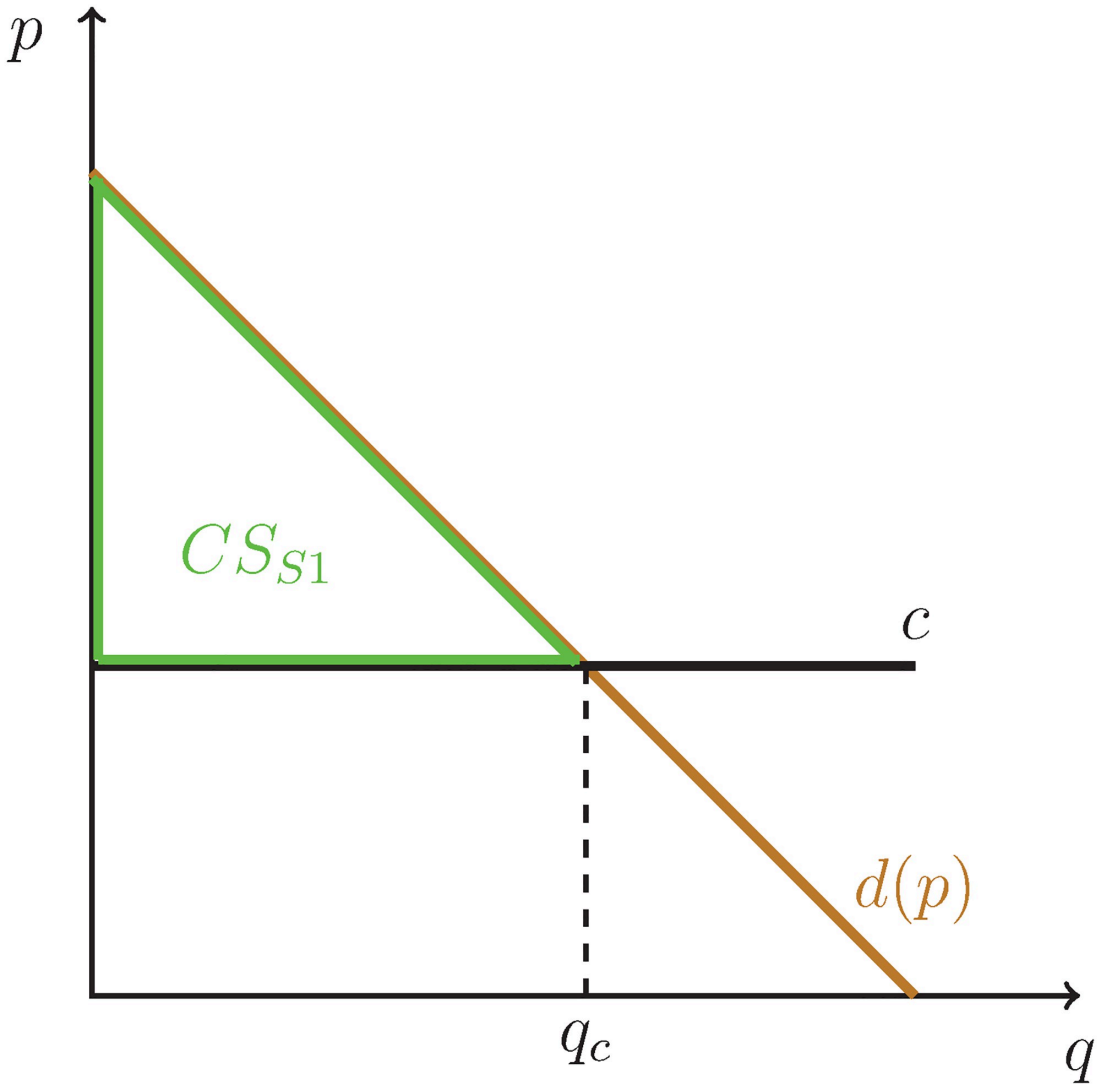
Case 2, market 1: Perfect competition with constant marginal cost.

In first market the innovation takes place. As in the first scenario, the product is produced at a constant cost *c* and there is no producer surplus as the price of the product is equal to the marginal cost. The market is illustrated graphically in Fig 3.

The demand curve is as in the first market in Case 1, the marginal cost is again constant at *c*:

D(q):p=ψ-ϕq.

The quantity produced with perfect competition is:

$$q_{c}=\frac{\psi -c}{\phi }.$$

The consumer surplus (the triangle below the demand curve and above the constant marginal cost *c*) is:

$$CS_{S1}=\frac{\left(\psi -c\right)}{2}q_{c}=\frac{\left(\psi -c\right)}{2}\frac{\left(\psi -c\right)}{\phi }=\frac{{\left(\psi -c\right)}^{2}}{2\phi }.$$

As there is no producer surplus in this market, the total welfare is equal to the consumer surplus:

$$TW_{S1}=CS_{S1}=\frac{{\left(\psi -c\right)}^{2}}{2\phi }.$$

#### Second market—Subsidized monopoly

**Fig 4:**
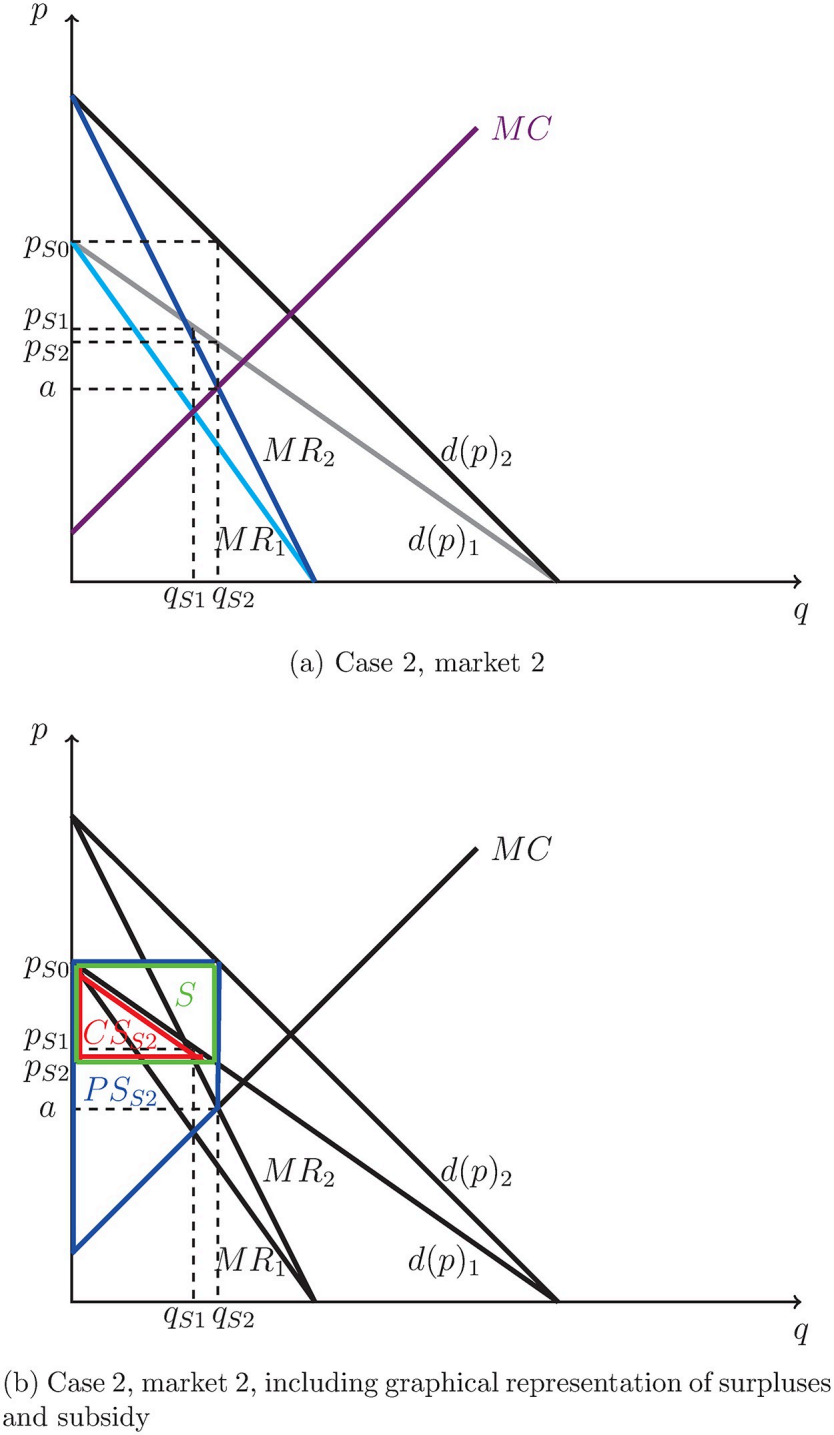
Case 2, market 2: Monopoly with increasing marginal cost and subsidy. (a) Case 2, market 2. (b) Case 2, market 2, including graphical representation of surpluses and subsidy.

There is a monopoly in the second market operating at increasing marginal cost, as in Case 1. However, now the originator receives a subsidy from the government. This subsidy takes the form of a fraction *m* of the price paid by consumers. It can be represented graphically by rotating the demand and marginal revenue curves, as shown in Fig 4 (a). The new demand curve *d*(*p*)_2_ now represents the demand by consumers at a price that already includes the subsidy (a higher quantity is demanded at each subsidy-inclusive price *x*, because the consumer only pays *x*/(1 + *m*)).

The equations for the marginal cost and for the original demand and marginal revenue curves are as in the second market of the first case in Case 1:

$$\begin{array}{cc} MC(q) & =\theta +\epsilon q\\ D{\left(q\right)}_{1}:p & =\gamma -\eta q\\ MR{\left(q\right)}_{1} & =\gamma -2\eta q. \end{array}$$

The demand and marginal revenue curves incorporating the subsidy are:

$$\begin{array}{cc} D{\left(q\right)}_{2}:p & =(\gamma -\eta q)(1+m)\\ MR{\left(q\right)}_{2} & =(\gamma -2\eta q)(1+m). \end{array}$$

The optimal quantity from the perspective of the monopolist is determined where marginal cost equals the subsidy-inclusive marginal revenue, which is indicated by *q*_*S*2_ in Fig 4 (a). The optimal price is the value determined by the demand curve for the respective quantity. The optimal quantity is given by:

$$\begin{array}{cc} \theta +\epsilon q & =(\gamma -2\eta q)(1+m)\\ \theta +\epsilon q & =\gamma -2\eta q+m\gamma -2m\eta q\\ \epsilon q+2\eta q+2m\eta q & =\gamma +m\gamma -\theta \\ q_{S2} & =\frac{(1+m)\gamma -\theta }{\epsilon +2\eta (1+m)}. \end{array}$$

The subsidy is paid by the government to the producer. The consumers thus face a price *p*_*S*2_ after the subsidy is introduced, (which is different from *p*_*S*1_, which would have been the price in the market without subsidy), lying on the original demand curve. The price that the originator receives includes the subsidy and thus lies on the subsidy-inclusive demand curve—it is denoted by *p*_*S*0_:

$$\begin{array}{c} p_{S0}=\left(\gamma -\eta q_{S2}\right)\left(1+m\right)=\left(\gamma -\eta \left(\frac{(1+m)\gamma -\theta }{\epsilon +2\eta (1+m)}\right)\right)\left(1+m\right). \end{array}$$

The price that the consumers are paying is *p*_*S*2_ and it is (1 + m) times smaller than the subsidy-inclusive price *p*_*S*0_:

$$p_{S2}=d_{1}\left(q_{S2}\right)=\frac{p_{S0}}{1+m}=\gamma -\eta \left(\frac{(1+m)\gamma -\theta }{\epsilon +2\eta (1+m)}\right).$$

The market, including the graphical representation of consumer surplus, producer surplus, and the paid subsidy is shown in Fig 4 (b). To calculate consumer and producer surpluses, we need the price at the intersection of MC with *MR*_2_, which we denote by *a*:

$$\begin{array}{c} a=\left(1+m\right)\left(\gamma -2\eta q_{S2}\right)=\left(1+m\right)\left(\gamma -2\eta \frac{(1+m)\gamma -\theta }{\epsilon +2\eta (1+m)}\right). \end{array}$$

The consumer surplus (the triangle below the original demand curve and above the price which consumers pay, *p*_2_) is:

$$\begin{array}{cc} CS_{S2} & =\frac{\left(\gamma -p_{S2}\right)}{2}q_{S2}=\frac{\left(\gamma -\left(\gamma -\eta \left(\frac{(1+m)\gamma -\theta }{\epsilon +2\eta (1+m)}\right)\right)\right)}{2}\left(\frac{(1+m)\gamma -\theta }{\epsilon +2\eta (1+m)}\right)\\ & =\frac{\eta }{2}{\left(\frac{(1+m)\gamma -\theta }{\epsilon +2\eta (1+m)}\right)}^{2}. \end{array}$$

The producer surplus (the area from 0 to the optimal quantity *q*_*S*2_ below *p*_*S*0_ and above the MC curve) is:

$$\begin{array}{cc} PS_{S2} & =\left(p_{S0}-a\right)q_{S2}+\frac{\left(a-\theta \right)}{2}q_{S2}=q_{S2}\left(p_{S0}-\frac{a}{2}-\frac{\theta }{2}\right)\\ & =\frac{(1+m)\gamma -\theta }{\epsilon +2\eta (1+m)}(\left(\left(\gamma -\eta \left(\frac{(1+m)\gamma -\theta }{\epsilon +2\eta (1+m)}\right)\right)\left(1+m\right)\right)\\ & -\frac{\left(1+m\right)\left(\gamma -2\eta \frac{(1+m)\gamma -\theta }{\epsilon +2\eta (1+m)}\right)}{2}-\frac{\theta }{2})\\ & =\frac{{\left(\left(1+m\right)\gamma -\theta \right)}^{2}}{2(\epsilon +2\eta (1+m))}. \end{array}$$

Finally, the government has to pay the subsidy. The total payment by the government is:

$$\begin{array}{cc} S & =\left(p_{S0}-p_{S2}\right)q_{S2}=\left(mp_{S2}\right)q_{S2}=m(\gamma -\eta \frac{(1+m)\gamma -\theta }{\epsilon +2\eta (1+m)})\frac{(1+m)\gamma -\theta }{\epsilon +2\eta (1+m)}\\ & =m\gamma \frac{(1+m)\gamma -\theta }{\epsilon +2\eta (1+m)}-m\eta {\left(\frac{(1+m)\gamma -\theta }{\epsilon +2\eta (1+m)}\right)}^{2}. \end{array}$$

Therefore, the total welfare in the second market is the sum of the consumer surplus and the producer surplus less the subsidy payment:

$$\begin{array}{cc} TW_{S2} & =CS_{S2}+PS_{S2}-S=q_{S2}\left(\frac{\gamma +p_{S2}-a-\theta }{2}\right)\\ & =\left(\frac{(1+m)\gamma -\theta }{\epsilon +2\eta (1+m)}\right)\left(\frac{\gamma +\left(\gamma -\eta \left(\frac{(1+m)\gamma -\theta }{\epsilon +2\eta (1+m)}\right)\right)-\left(1+m\right)\left(\gamma -2\eta \frac{(1+m)\gamma -\theta }{\epsilon +2\eta (1+m)}\right)-\theta }{2}\right)\\ & =\frac{(1+m)\gamma -\theta }{\epsilon +2\eta (1+m)}*\frac{\left(1-m\right)\gamma +\left(1+2m\right)\eta \frac{(1+m)\gamma -\theta }{\epsilon +2\eta (1+m)}-\theta }{2}. \end{array}$$

## Theoretical results

We first show the results on social welfare and producer surplus for both policy regimes (cases) separately. Then we combine these formulas to allow for a comparison of the two cases. Thus, we calculate the difference in social welfare (from one case to the other) and the difference in producer surplus. The formulas follow directly from the derivations in the previous section.

The results concern two dimensions. The first one is social welfare. From the government’s perspective, the policy regime with higher social welfare should be preferred. The second dimension is the producer surplus. If, in addition to social welfare, also the producer surplus is higher in the regime without intellectual property rights but with a subsidy in the second market, then abolishing intellectual property protection while introducing the subsidy would be beneficial not only from a societal point of view, but even from the point of view of the originator. If the originator is also better off under the subsidy regime, he or she also has an incentive to keep being creative, so that one can be sure that the creative works keep being produced. In some cases, one may also be interested to merely calculate the social welfare or producer surplus in one case, which is why we state these separately for the two cases as well (potentially, somebody may be interested in comparing social welfare or producer surplus of different industries—e.g., the music industry versus visual arts—within the same policy regime).

### Results on total welfare and producer surplus for both cases separately

The total welfare arising in both markets jointly in Case 1 (status quo) is as follows:

$$\begin{array}{c} TW_{M}=TW_{M1}+TW_{M2}=\frac{3{\left(\psi -c\right)}^{2}}{8\phi }+\frac{{\left(\gamma -\theta \right)}^{2}}{2(\epsilon +2\eta )}\left(1+\frac{\eta }{\epsilon +2\eta }\right). \end{array}$$

The producer surplus in both markets jointly in Case 1 (status quo) is:

$$\begin{array}{c} PS_{M}=\frac{{\left(\psi -c\right)}^{2}}{4\phi }+\frac{{\left(\gamma -\theta \right)}^{2}}{2(\epsilon +2\eta )}. \end{array}$$

The total welfare from both markets jointly in Case 2 (subsidy regime) is:

$$\begin{array}{cc} TW_{S} & =TW_{S1}+TW_{S2}\\ & =\frac{{\left(\psi -c\right)}^{2}}{2\phi }+\frac{(1+m)\gamma -\theta }{\epsilon +2\eta (1+m)}*\frac{\left(1-m\right)\gamma +\left(1+2m\right)\eta \frac{(1+m)\gamma -\theta }{\epsilon +2\eta (1+m)}-\theta }{2}. \end{array}$$

The total producer surplus in Case 2 (subsidy regime) is:

$$\begin{array}{c} PS_{S}=PS_{S2}=\frac{{\left(\left(1+m\right)\gamma -\theta \right)}^{2}}{2(\epsilon +2\eta (1+m))}. \end{array}$$

### Comparison of the cases

The following formulas allow for a comparison of the two policies. The first formula shows the difference in total welfare between both cases. From the point of view of social welfare, it is desirable to replace intellectual property rights in the first market with a subsidy of size *m* in the second market if the below difference is positive:

$$\begin{array}{cc} TW_{S}-TW_{M} & =\frac{{\left(\psi -c\right)}^{2}}{8\phi }+\frac{(1+m)\gamma -\theta }{\epsilon +2\eta (1+m)}*\frac{\left(1-m\right)\gamma +\left(1+2m\right)\eta \frac{(1+m)\gamma -\theta }{\epsilon +2\eta (1+m)}-\theta }{2}\\ & -\frac{\eta }{2}{\left(\frac{\gamma -\theta }{\epsilon +2\eta }\right)}^{2}-\frac{{\left(\gamma -\theta \right)}^{2}}{2(\epsilon +2\eta )}. \end{array}$$

If not only social welfare is higher in case 2 than in case 1, but also producer surplus, then the switch from intellectual property protection in the first market to subsidy of size *m* in the second market would even be preferred by the originator. This is the case when the following difference is positive:

$$\begin{array}{c} PS_{S}-PS_{M}=\frac{{\left(\left(1+m\right)\gamma -\theta \right)}^{2}}{2(\epsilon +2\eta (1+m))}-\frac{{\left(\psi -c\right)}^{2}}{4\phi }-\frac{{\left(\gamma -\theta \right)}^{2}}{2(\epsilon +2\eta )}. \end{array}$$

## Application example

Here, we will show a numerical example of how the framework can be applied. We take the music industry with records as the first market and live performances as the second market. As the status quo, we take the case of intellectual property protection (IPP) in the first market, without subsidy in the second market (Case 1). We then consider the implications for social welfare and producer surplus for different values of the subsidy *m* in the second market, when switching to a regime without intellectual property right protection in the first market (Case 2).

For many parameters of the model, thorough parameter estimates are missing in the empirical literature. Therefore, the parameters that we choose are (where they are not informed by the empirical literature), guesses of what could be reasonable values.

In the first market, we use the following specification. For simplicity (and because it is appropriate in the market for digital records), we assume the cost of producing additional copies of a record to be zero. We choose a demand function with parameters *ψ* = 2 and *ϕ* = 1. This specification leads to a sold quantity of one in the status quo (such a normalization is unproblematic—this is just a change of the unit in which quantity is measured), a price of one, and a demand elasticity of (negative) one. The value of the demand elasticity is close to values found in the empirical literature (see [19] or the discussion in [20]).

In the second market, we use the following specification. We again normalize the quantity in the status quo to one (again, this is just a change in the unit of measurement; this means that the price in the status quo must in general be different from one to allow the markets to differ in terms of size). The parameters *γ*, *η*, *θ*, and *ϵ* need to be determined (these parameters need to satisfy the equation *γ* − *θ* = *ϵ* + 2*η*, due to the normalization of the quantity in the status quo to one). We use a slope of the (inverse) demand curve of *η* = 0.2, which is lower than the slope of the demand curve in the first market. That is, changes in price lead to relatively larger changes in demanded quantity (this seems like a reasonable assumption, but it is not based on rigorous econometric estimation). We use a marginal cost curve starting at a price *θ* = 1, signifying that performing live already comes at a cost, even if only very few live performances are held (equipment, etc.). We also choose a relatively steep marginal cost curve in this market, *ϵ* = 3, as time of artists is extremely limited (giving just a few concerts a month may be little burden for artists, but it is extremely costly for them to do more than a few per week). These numbers lead (with above normalization of the quantity) to a parameter *γ* = 4.4, which indicates the intersection of the demand curve with the vertical axis. Together, this means that the price for the status-quo quantity of live performances is about four times the price for the status-quo quantity of records in the first market. This is in line with the findings of [4], who shows that live performances make up for a much larger share of an artist’s revenue than record sales.

**Fig 5:**
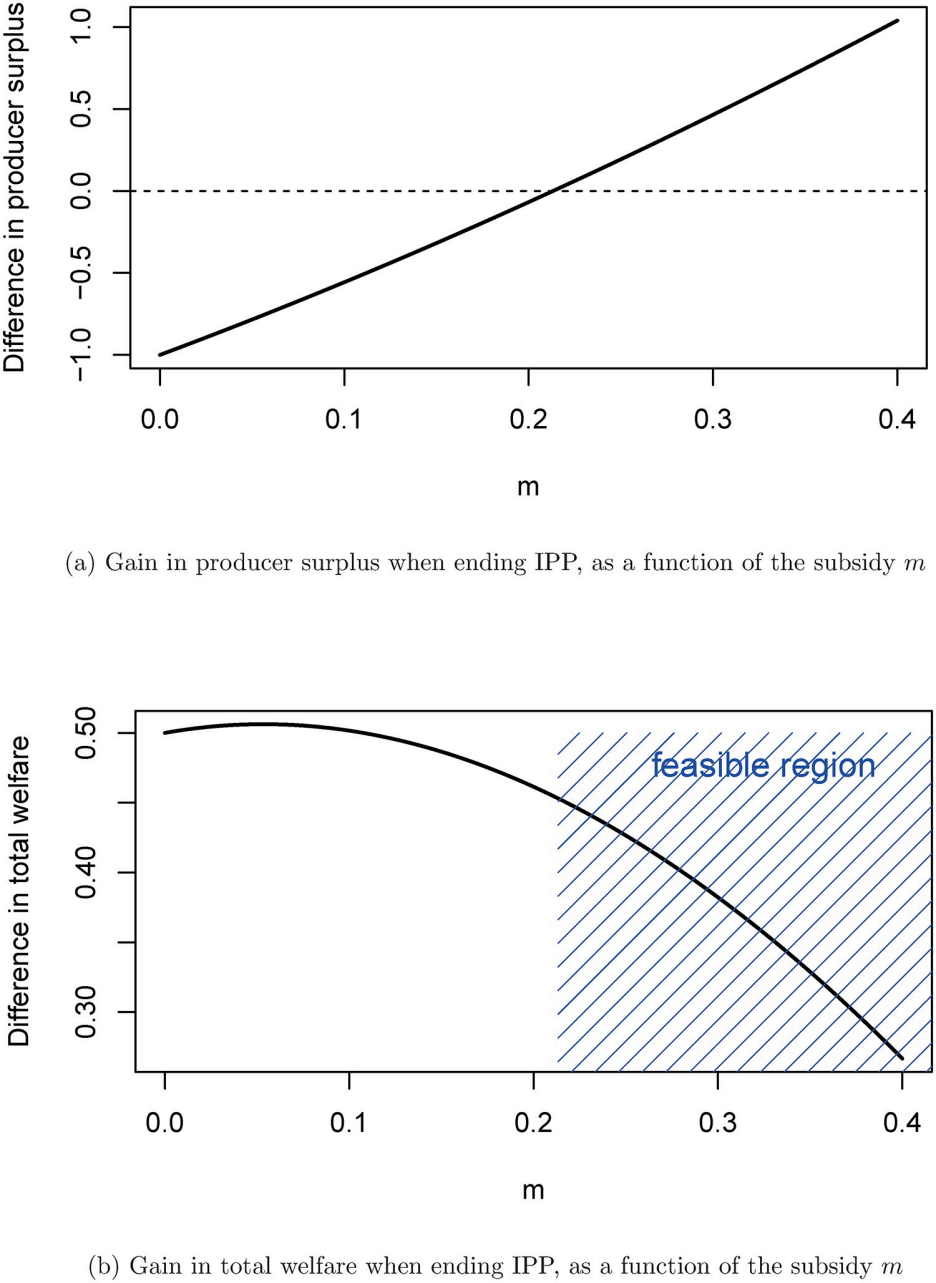
Producer surplus and total welfare when ending IPP, in dependence of the subsidy. (a) Gain in producer surplus when ending IPP, as a function of the subsidy *m*, (b) Gain in total welfare when ending IPP, as a function of the subsidy *m*.

Fig 5 (a) shows the difference in producer surplus when changing the regime to one without intellectual property protection but with subsidies in the second market. The difference in consumer surplus in shown in dependence of the height of the subsidy *m*. Negative numbers signify that producer surplus is higher with intellectual property protection. Fig 5 (b) shows the changes in total welfare, again in dependence of *m*.

The function in Fig 5 (a) naturally increases in *m*. The higher the subsidy, the better for the artist (the producer of music), when the regime is such there is a subsidy in the second market. However, whether one regime is better than the other (for the artist) depends on the value of *m*. Here, the difference in producer surplus is zero at about *m* = 0.21. That is, for subsidies above about 21%, the artist is better off in the regime with the subsidy, for subsidies below this value, the artist would be better off without subsidy but therefore with intellectual property protection in the first market.

Fig 5 (b) shows that total welfare would be highest (all else equal), when switching to the regime with a subsidy in the second market for a value of the subsidy of only about five percent. Social welfare is already higher than in the other regime with a subsidy of zero (because of the gains for consumers when there is perfect competition for the consumer). When increasing the subsidy from zero, total welfare first increases slightly (because of the incentive for the artist to produce above the zero-subsidy monopoly level in the second market, which weighs stronger than the cost of the subsidy at low levels of the subsidy), but at some point, total welfare starts to decrease, because of the deadweight loss associated with an excessive subsidy. In this example, the gain of total welfare from switching to the regime with a subsidy would turn negative for subsidies above around 57%.

Note that, in this partial equilibrium model, it is assumed that the artist always produces the music. Taking the status quo of intellectual property protection and switching to the different regime with a sufficiently high subsidy, so that the artist is better off, it can reasonably be assumed that the artist will keep producing the music. However, when there is a switch to the new regime with a subsidy that would make the artist worse off (conditional on him or her producing the music), it is not clear that the artist still has sufficient incentives to produce the music. Only those values of *m*, for which the artist is not worse off under the new regime are therefore certain to lead to the needed music production, so that the gains in total welfare can be realized. We highlight this area, which we call the feasible region, in Fig 5 (b).

Putting the absolute numbers on the vertical axes of the graphs, into perspective, producer surplus and total welfare in the status quo are at 2.7 and 3.3, respectively. That is, in the status quo with intellectual property protection, the producer surplus makes up for about 80% of total welfare. When switching to the new regime with a subsidy of 22% to 25% (these values are in the feasible region), producer surplus would increase moderately by up to about seven percent. Total welfare would increase by about 13%.

## Discussion

In applications, which regime and what level of subsidy are best will necessarily depend on the (estimated) parameters. This depends on the slopes of the curves, but also on the levels—if the second market is very small in comparison to the first, it is less likely that the introduction of a (reasonably sized) subsidy can compensate for the loss of intellectual property right protection in the first market (correspondingly, larger secondary markets make it more likely that replacing intellectual property rights in the first market with subsidies in the second is a good idea). In the numerical application example to the provision of music in the records and live performance markets, we find that the subsidy regime (Case 2) may indeed be better than the status quo (Case 1) if the subsidy in the market for live performances is sufficient.

The main difference of our policy implications to the implications in much of the literature is the following. While subsidies or rewards are often superior to intellectual property rights in the literature, these are always subsidies or rewards in the same market in which the innovation takes places. The exploitation of the secondary market in which the originators operate is unique to our study. The secondary market allows for subsidies that can be very easily implemented, for example with the voluntary subsidy scheme that we outline. The reason why the subsidy we propose can more easily be implemented than a subsidy in the market in which the innovation takes place is that for a subsidy in the primary market one would need to measure the quality of the innovation in some way after intellectual property rights have been removed, which seems to be a very complex and difficult endeavor (in our case, this is not needed, because the sales in the secondary market are observed).

In our numerical application example, we find that the subsidy scheme would be better than the status quo if the subsidy were about 22% or higher (and below about 57%), assuming some reasonable parameter values. Unfortunately, the basis on which the calibration of the parameters is founded does not consist in thorough econometric estimates of these parameters, as such parameters hardly exist (note, however, that whether the exact turning point when the subsidy regime becomes the superior policy regime is around 22%, as in our example, or around 5, 10, 15, 20, 25, or 30% does not change the conclusions qualitatively). The non-availability of such estimates thus limits the applicability of our model. Unfortunately, economically simple concepts like demand and marginal revenue equations need to be estimated with non-trivial econometric techniques, because they usually cannot be observed. Doing so would be beyond the scope of this paper.

## Conclusion

Assigning intellectual property rights means assigning monopoly rights, and these automatically come with a welfare loss. Therefore, scholars have long been suggesting to use reward schemes instead of assigning monopoly rights. However, these suggestions have not considerably affected actual policy yet. A reason may be that implementing such theoretically beneficial reward schemes seems to be difficult in practice. We propose an easy way to implement rewards, which can be done when there is a second market in which originators operate. It can be better for social welfare and even for originators themselves if there is a subsidy in the second market instead of monopoly rights in the first market. In countries with a high value-added tax, such a subsidy could be introduced particularly easily by granting a reduced or zero VAT rate in the second market. We have provided a first microeconomic partial-equilibrium framework formalizing this idea.

In a numerical example with values that seem appropriate for the music industry, we calculate that removing intellectual property rights in the records market and therefore subsidizing live performances with a subsidy of 22% to 25% would make artists moderately better off and increase total welfare considerably. Given that VAT rates in several countries are between 20% and 25%, it may in some countries already be sufficient to exempt live performances from VAT.

The introduction of a subsidy in the second market while removing IPP protection in the first market may be particularly popular (and thus politically feasible) if it is not applied to everyone in an industry automatically. Instead, it could be implemented as a voluntary scheme. Originators may choose to be part of the subsidy program in the second market when they commit to making their works freely available in the first market. As the participation in the program would be voluntary, such a subsidy scheme could be easily implemented, even without changing the laws on intellectual property rights.

Our model can be most successfully applied when high-quality estimates of the parameters of the marginal revenue and demand functions are available. Unfortunately, such estimates are only rarely available for the creative or innovative industries. Empirically estimating these demand and marginal revenue functions is a challenging task (because demand and marginal revenues can usually not be observed), but it potentially constitutes an interesting avenue for future research.

## Supporting information

S1 File R-script. This file contains the R-code to produce the results of the application example. The code is annotated.
(R) Click here for additional data file.

## References

[1] Boldrin M, Levine DK. Against Intellectual Monopoly. Cambridge University Press; 2008.

[2] Bessen J, Maskin E. Sequential innovation, patents, and imitation. The RAND Journal of Economics. 2009;40(4):611–635. doi: 10.1111/j.1756-2171.2009.00081.x

[3] Biasi B, Moser P. Effects of copyrights on science: Evidence from the WWII book republication program. American Economic Journal: Microeconomics. 2020;.

[4] DiCola P. Money from music: Survey evidence on musicians’ revenue and lessons about copyright incentives. Arizona Law Review. 2013;55:301.

[5] Liu J. Copyright for Blockheads: An Empirical Study of Market Incentive and Intrinsic Motivation. Columbia Journal of Law & the Arts. 2014;38:467.

[6] Perrenoud M, Bataille P. Artist, craftsman, teacher:“being a musician” in France and Switzerland. Popular Music and Society. 2017;40(5):592–604. doi: 10.1080/03007766.2017.1348666

[7] Litman J. What We Don’t See When We See Copyright as Property. The Cambridge Law Journal. 2018;77(3):536–558. doi: 10.1017/S0008197318000600

[8] Wright BD. The economics of invention incentives: Patents, prizes, and research contracts. The American Economic Review. 1983;73(4):691–707.

[9] Shavell S, Van Ypersele T. Rewards versus intellectual property rights. The Journal of Law and Economics. 2001;44(2):525–547. doi: 10.1086/322811

[10] Gandjour A, Chernyak N. A new prize system for drug innovation. Health Policy. 2011;102(2–3):170–177. doi: 10.1016/j.healthpol.2011.06.001 21724290

[11] Grinols EL, Lin HC. Patent replacement and welfare gains. Journal of Economic Dynamics and Control. 2011;35(9):1586–1604. doi: 10.1016/j.jedc.2011.04.010

[12] Chari VV, Golosov M, Tsyvinski A. Prizes and patents: Using market signals to provide incentives for innovations. Journal of Economic Theory. 2012;147(2):781–801. doi: 10.1016/j.jet.2011.04.004

[13] Alcalá F, González-Maestre M. Copying, superstars, and artistic creation. Information Economics and Policy. 2010;22(4):365–378. doi: 10.1016/j.infoecopol.2010.05.001

[14] Davis L. Intellectual property rights, strategy and policy. Economics of Innovation and New Technology. 2004;13(5):399–415. doi: 10.1080/1043859042000188683

[15] Brunt L, Lerner J, Nicholas T. Inducement prizes and innovation. The Journal of Industrial Economics. 2012;60(4):657–696. doi: 10.1111/joie.12002

[16] Stiglitz JE. Economic foundations of intellectual property rights. Duke Law Journal. 2008;57:1693.

[17] Clancy MS, Moschini G. Incentives for innovation: Patents, prizes, and research contracts. Applied Economic Perspectives and Policy. 2013;35(2):206–241. doi: 10.1093/aepp/ppt012

[18] Abramowicz M. Prize and reward alternatives to intellectual property. In: Research Handbook on the Economics of Intellectual Property Law. Edward Elgar Publishing; 2019.

[19] Stevans LK, Sessions DN. An empirical investigation into the effect of music downloading on the consumer expenditure of recorded music: A time series approach. Journal of Consumer Policy. 2005;28(3):311–324. doi: 10.1007/s10603-005-8645-y

[20] Asai S. Demand analysis of hit music in Japan. Journal of Cultural Economics. 2011;35(2):101–117. doi: 10.1007/s10824-011-9139-1

